# From welfare indicator to welfare contributor: the role of play in building flexibility and resilience in captive animals

**DOI:** 10.1098/rspb.2025.1962

**Published:** 2025-11-26

**Authors:** Amelia St John Wallis, Michael T. Mendl, Benjamin Lecorps, Suzanne D. E. Held

**Affiliations:** ^1^ Bristol Veterinary School, University of Bristol, Bristol, UK

**Keywords:** animal well-being, coping, captivity, adaptation, animal play, behavioural plasticity

## Abstract

Multiple behavioural indicators have been explored to understand how captive individuals feel about certain environments and events, one of which is play. However, play likely also contributes to enhancing future welfare. A key pathway linking play and welfare may lie in the development of flexibility and resilience, which enable individuals to adapt and manage the inevitable challenges they will experience. This article develops a framework for how play contributes to captive animal welfare through flexibility and resilience by synthesizing theories from multiple fields, including the training for the unexpected theory, the broaden-and-build theory of positive emotions and the cognitive bias literature. Findings from multidisciplinary studies, including those assessing the correlates of the playfulness trait in humans, the effect of play interventions on flexibility in children and the impact of social play deprivation in rodents, provide preliminary support for these play–flexibility–resilience relationships. We provide recommendations for future approaches to the play–welfare relationship in captive animals, including exploring how play can be used to enhance long-term welfare, the welfare impact of play in adult versus young animals and the differences between play types, and whether and how the playfulness trait contributes to welfare across species.

## 1. Introduction

Play is a common feature of the juvenile period across multiple species [1,2] and is also observed in adult animals [3]. The types of play are multifaceted, including locomotor play, which involves a form of movement (e.g. running), social play, which often involves play-fighting with a conspecific, and object play, where play behaviours are directed towards manipulating inanimate objects. One key part of Burghardt’s definition of play [4] (table 1) suggests that it is commonly observed when the individual is not subject to stress or other urgent motivations (e.g. hunger or thirst). On the assumption that suppressed play indicates greater stress and a more negative affective state, and with recent perspectives defining animal welfare as ‘the balance of positive and negative affective states’ [9] (table 1), play levels have thus often been used as a welfare indicator [1,2]. However, space should also be given to how play may not only indicate but contribute to affective welfare outcomes.

**Table 1.** Definitions of key terms*.*

term	definition
play	play is repeated, self-rewarding and incompletely functional behaviour differing from fully functional versions structurally, contextually or ontogenetically and most commonly performed when the animal is in a relaxed setting free of threats to its biological fitness [4]
animal playfulness trait	an underlying trait that stably influences an individual’s propensity to play across time and contexts [5,6]
affective state	a state of experience that has a positive or negative valence (i.e. rewarding/pleasant, aversive), including components of emotion (a short-term valenced state elicited by a stimulus), mood (a longer-term valenced state not elicited by a specific stimulus) and sensory and homeostatic experiences (e.g. pain, nausea, thermal comfort) [7,8]
animal welfare	‘the balance of positive and negative affective states over the period of time of interest, which can span from hours to years to lifelong’ [9]
flexibility	the ability to change established behavioural responses and produce varied actions when faced with new challenges and situations [10]
resilience	an individual’s ability to adaptively respond to and effectively recover from stressors [11–13]
cognitive bias	relative differences in the processing of environmental stimuli caused by current or past affective states [14,15]

In the short term, it is easy to conceive that play is rewarding in humans (for a review, see [16]), and it has also been suggested to be pleasurable in non-human animals (hereafter referred to as ‘animals’ [17,18]). For example, in previous studies, rats developed a conditioned place preference for an area associated with social play [19], and tickling by an experimenter, designed to simulate social play, increased cognitive bias markers consistent with positive affect [20]. Therefore, play does not only reflect but also positively influences an individual’s affective state in the moment [20–22]. Play behaviour may also serve as a stress coping mechanism in human children [23] and animals [24,25] (for reviews, see [1,26]) through supporting the individual to balance their positive and negative affect [27,28].

Since play carries significant costs in the wild (e.g. increased predation and injury risk, energy costs) [29,30], evolutionary theories suggest that this behaviour is also likely to provide both immediate and future benefits to the individual that ultimately outweigh them. For example, immediate benefits include generating positive emotion [17–20], hypothesized to reward playing in juveniles for whom the fitness benefits are typically delayed. Future benefits may involve refining foraging, motor and social skills from playing during development [3,30–33]. Social play may also relieve social tension, especially in adult animals (for reviews, see [26,34]). One notable pathway through which play may influence future welfare is through the development of flexibility and resilience (see table 1 for definitions), a crucial determinant of welfare given the challenges faced by captive animals [35,36]. Flexibility, especially ‘behavioural flexibility’, has been conceptualized in numerous ways across experimental psychology and behavioural ecology [37] but is typically defined as the ability to change established behavioural responses and produce varied actions when faced with new challenges and situations [10] (table 1). Flexibility may enhance resilience by enabling the individual to successfully adapt to changing situations or challenges that can be overcome by implementing a new behavioural strategy (for reviews, see [38–40]). Flexibility is related to self-reported resilience in humans [41–44], and this is backed by experimental evidence in animals. For example, calves with better reversal learning (i.e. cognitive flexibility) showed better adaptation to a new environment [45], and mice with better reversal learning showed more positive emotional outcomes following chronic social defeat stress [46]. However, it should also be noted that an individual’s ability to manage some challenges in captivity, such as barren, unchanging environments, may not be significantly improved by being more flexible.

A key evolutionary theory linking play to flexibility and resilience is the ‘training for the unexpected’ theory [39], which suggests that play pushes the individual to place itself in a variety of disruptive and difficult situations that they must learn to resolve appropriately [39,47], providing opportunities to develop flexibility in their behavioural responses when faced with situations to overcome later in life [3,39]. Play often involves a self-handicapping component, whereby the individual places themselves in a compromising situation (e.g. the unusual physical movements of locomotor play [39]; reciprocity in social play fighting in rats [33]), which may help them to learn to flexibly resolve difficulties and develop positive expectations about their ability to manage them.

A more recent theory suggested that play may have evolved as a byproduct of intrinsic exploration (i.e. information-seeking motivated by curiosity rather than predetermined goals), with both behaviours enabling the flexible adaptation of behaviour in response to change [48]. Relatedly, Bayesian and predictive processing theories suggest that living entities seek to reduce their uncertainty arising from high prediction errors and, thus, aim to improve the predictions they make about the environment [49,50], meaning individuals are likely motivated to explore to gather information about their sensorimotor environment and how it works [51,52]. Play may support the individual to gain information about themselves, their body and its capabilities, the actions of others and how they can interact with the world, which can then be generalized to similar objects or situations in the future [16]. Ultimately, such information-gathering would be beneficial for developing different responses that could be employed across situations, potentially supporting flexible behaviour.

Since engaging in play likely produces positive affect [1,2,18], an interesting link also exists between play and flexibility through the lens of the broaden-and-build theory of positive emotions from human psychology [53–55]. This theory suggests that positive affective experiences broaden ‘thought–action repertoires’ (i.e. the range of possible actions and thoughts generated in response to varying situations), enabling the individual to think and act in a more flexible way when responding to challenges [53,54]. Positive emotions in humans enhance the use of global (i.e. wider) versus local (i.e. narrower) visual processing when judging pattern similarities [55], widen attentional scope in visuospatial tasks [56] and increase the number of actions generated in response to a given situation [55]. Positive emotional states in horses improved extinction learning (i.e. how fast they stopped responding to a cue that was no longer being rewarded), indicating improved cognitive flexibility [57]. Conversely, rats experiencing chronic pain were more perseverative when a cue was no longer rewarded [58], indicating that the negative affective state induced by pain may have decreased cognitive flexibility. However, these two animal studies did not explore attentional scope. A proof-of-concept study in cows aiming to assess attention to local versus global pattern similarities reported that individuals in the assumed negative condition (i.e. part-time versus full-time calf contact) never approached the images requiring global versus local processing, which the authors interpreted as being suggestive of a narrower attentional scope in this group [59].

Relatedly, the cognitive bias literature (see table 1 for a definition of ‘cognitive bias’) suggests that a positive affective state, such as that caused by play, may alter perceptions of possible rewards in the environment and the openness of an individual’s responses to them [7,15]. For example, in judgement bias tests (JBTs), individuals in negative affective states make more negative (pessimistic) responses to ambiguous reward cues, indicating lower reward expectations and/or lower perceptions of reward value (for reviews, see [7,15,36]; meta-analyses, see [60,61]) and the use of safer, more conservative response patterns [54]. In contrast, those in positive affective states tend to respond in a more positive (optimistic) way [15,62], indicating greater willingness to sample more varied information and openness to potential risks and rewards [53,54]. As such, an individual with positive experiences from play may be more open to engaging in a wider range of actions across situations despite potential risks, allowing them to respond more flexibly to the environment.

Positive affect from play may also directly impact resilience through reducing negative cognitive biases. One framework of human resilience [12] suggests that a positive appraisal style, supported by positive affective experiences [7,54], is key for generating a more resilient phenotype. In humans, negative cognitive biases are related to poorer coping with stressors and vulnerability to mood disorders [63–66]. In animals, pessimistic responding on JBTs (i.e. negative judgement bias) is also related to greater anhedonic-like responses to stressors measured using sucrose consumption, suggestive of poorer resilience [67]. For example, pessimistic judgement biases have been shown to predict greater anhedonic-like responses to chronic stress in rats [68] and disbudding in calves [69]. Conversely, positive cognitive biases and the consequent openness to rewards may increase the individual’s chance to have further positive experiences, creating a feedback loop of affective state, not dissimilar to Fredrickson’s upward spiral of emotional wellbeing in humans [70].

These relationships between play, positive affect, flexibility and resilience are highlighted in the model in box 1, where play (i) provides experiences that support the development of flexible behaviour; (ii) supports flexibility and resilience through enhancing positive affect; and (iii) induces positive cognitive biases through enhancing positive affect, which have expected positive effects on flexibility and resilience. Play levels are considered to be influenced by the play opportunities afforded to the individual by their environment (which are open to intervention), their *a priori* playfulness trait (see definition in table 1) and their play motivation, which may vary over time (e.g. in response to positive affective experiences, as shown in the model [1,2,5]) and due to other individual characteristics (e.g. playfulness trait, boldness and fearfulness; e.g. [74]). Indeed, playfulness as a trait has been suggested to exist in certain animals (e.g. dogs [75], rats [76], mice [77], cats [78], pigs [79] and horses [80]). These three influences are not viewed as independent, as the playfulness trait may influence play motivation, and play motivation and opportunities may reciprocally influence each other. Since resilience allows the individual to adaptively respond to and recover from stressors, the model also highlights its reciprocal influence on positive affective state.

Box 1: **Model of the proposed play–flexibility–resilience relationships.**

**Figure 1.**
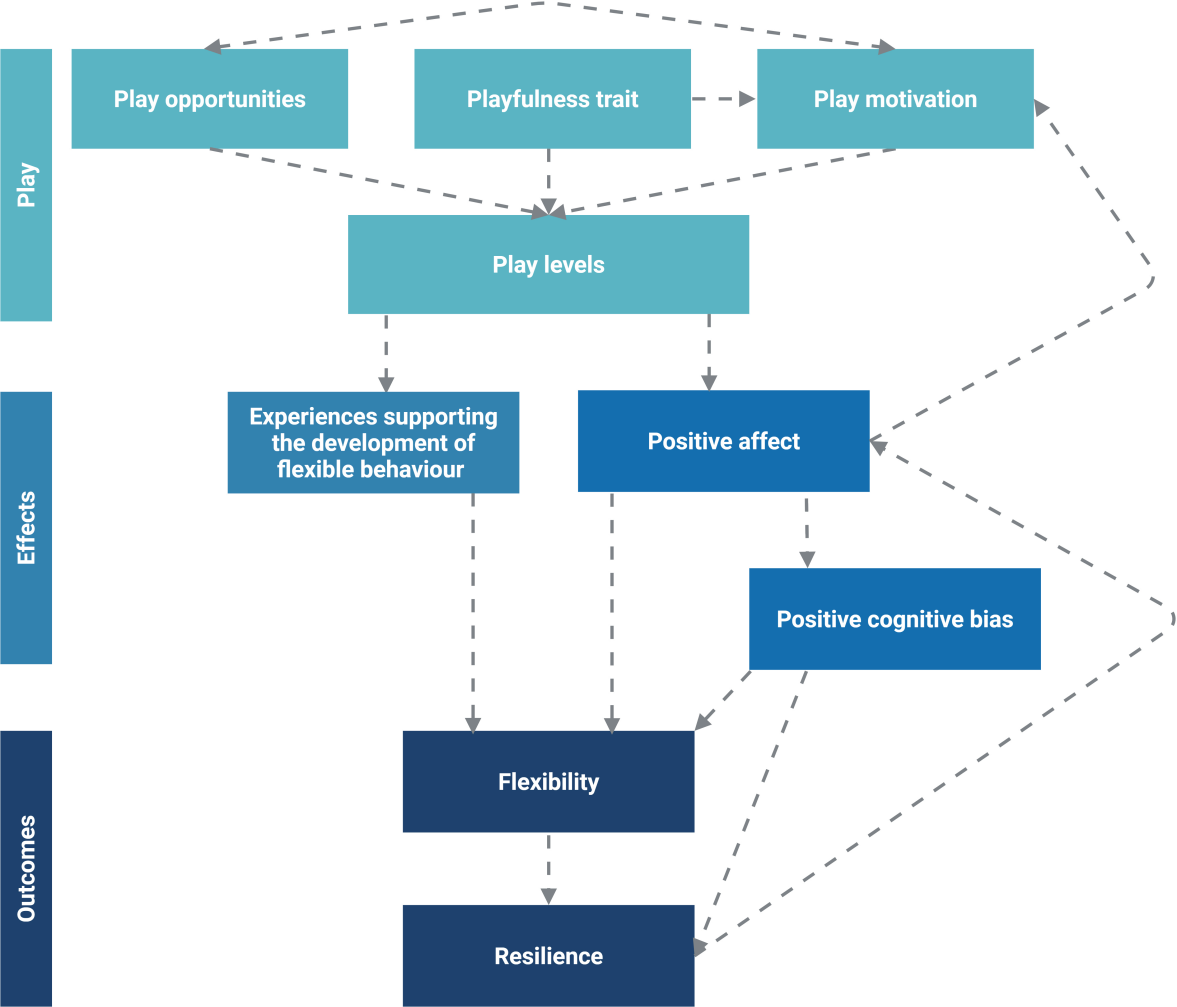
Model illustrating the potential pathways through which play may influence the development of flexibility and resilience (created in BioRender.com).

The theories reviewed here suggest multiple pathways through which play may influence the development of flexibility and resilience (figure 1). The model does not distinguish between play types (e.g. social versus locomotor versus object play), although their contributions to the outcomes may differ due to their varying nature and potential differences in motivational underpinnings [71–73]. There is, however, a distinction made between ‘play levels’ and the ‘playfulness trait’; the former refers to the amount of play an individual shows in a specific context, whereas the latter suggests the presence of an *a priori* trait underpinning differences in individual playfulness across contexts and time (see table 1 for a definition).

If these play–flexibility–resilience relationships hold true, restricted play opportunities in intensive management systems (e.g. individual housing and small pens in dairy calves) [81] may represent a key area of vulnerability for captive animals. Therefore, this review examines the current experimental evidence linking play to the development of flexibility and resilience in humans and animals and presents recommendations for future research exploring the role of play as a welfare contributor and utilizing play opportunities to improve welfare. Although the functions of play may vary across development, this review explores evidence for the play–flexibility–resilience relationships in both juvenile and adult animals. Finally, the contributions of each of the different play types (i.e. locomotor, social and object play) to flexibility and resilience are not distinguished (for further discussion, see §3a(i)).

## 2. Experimental evidence for the play–flexibility–resilience relationship

This section reviews studies highlighting the relationships between play, flexibility and resilience. Studies looking at the relationships between playfulness as a trait and flexibility and resilience have predominantly been conducted in humans, for whom playfulness has been discussed as an enduring and multi-dimensional personality trait (e.g. [82–84]). However, human playfulness has been described and measured in varying ways. One model defined playfulness as a characteristic that ‘allows people to frame or reframe everyday situations in a way such that they experience them as entertaining, and/or intellectually stimulating, and/or personally interesting’ [82], whereas another [83] suggested that playfulness includes fun-seeking motivation, uninhibitedness and spontaneity. It is not clear how well the findings of studies using these self-reported playfulness scores from humans can be applied to ‘playfulness’ as a trait in animals (table 1), which should be assessed based on their propensity to play across situations and time, in line with the animal personality literature [85].

### (a) Play as a contributor to flexibility

#### (i) Playfulness trait and flexibility

For flexibility, one study in humans found that self-reported ‘playfulness’ on the adult playfulness trait scale (APTS), which includes fun-seeking motivation, uninhibitedness and spontaneity, was positively correlated with self-reported adaptability to challenging situations (e.g. learning new knowledge, managing stress and uncertainty) [86]. Although self-report bias should be considered, combining this finding with the line of work suggesting that the human playfulness trait supports the use of flexible coping strategies and thus improves resilience [6,87], it seems that, in humans at least, playfulness may have some beneficial effect on flexibility development.

Relatedly, a study in 6- to 7-year-old children investigated the relationship between cognitive flexibility on the dimensional change card sort task (where the player must flexibly switch between card-sorting rules [88]) and teacher-measured play characteristics [89]. There was a positive relationship between play leadership (i.e. taking the lead over peers during play) and play conformity (i.e. following others’ rules during play) and cognitive flexibility [89]. These findings suggest a role for certain elements of social play, such as leading and compromising with peers, in the development of flexibility. However, animal studies assessing the relationships between playfulness, characteristics of play behaviour and flexibility outcomes are lacking.

#### (ii) Play levels and flexibility

Concerning play levels, previous studies have reported that rats isolated during the juvenile period to limit social play showed greater behavioural rigidity in response to reward contingency reversal [90] and used a simpler strategy involving perseverative rather than feedback-based responding during reversal learning, suggesting poorer cognitive flexibility [91]. In the latter study, this detrimental impact on cognitive flexibility was partially rescued by providing 1 h of social play time per day, addressing some of the concerns regarding the confounding effect of social isolation in social play deprivation studies.

This study also identified reduced medial prefrontal cortex (mPFC) inhibitory synaptic signals in the play-deprived rats [91]. The mPFC is involved in the top-down control of other neural circuits, which is important for flexibility through supporting the inhibition of inappropriate responses [92]. Therefore, the reduced mPFC inhibitory signals may have affected the deprived rats’ ability to stop previous responses and try a new strategy, reducing their flexibility [91]. Indeed, social play is important for learning ‘when to stop’ to prevent playful interactions from turning into aggression [32]. The differences in inhibitory neural activity observed may have been related to the impact of play on dendritic pruning, as being able to play enhances dendritic pruning in frontal areas [93,94]. However, in [91], the reduction in inhibitory signals was not rescued by a daily 1 h of play, suggesting either that this change was a general effect of social isolation or that the amount of play provided was not enough for normative neuronal development. Additionally, since cognitive flexibility was partially rescued by play provision, mPFC inhibitory signals may not be the sole contributor to flexibility skills. Another line of evidence linking play to flexibility through neuronal mechanisms comes from a study showing that play availability enhances the plasticity of mPFC neurons to later experiences (i.e. greater changes in neuron morphology after nicotine administration) [95]. Therefore, the conditions allowing for juvenile play may also enable the brain to be more plastic.

Overall, these findings highlight a role for social play opportunities in developing flexibility and indicate that neural mechanisms (i.e. the development of mPFC inhibitory control, frontal dendritic pruning and plasticity) may be involved in this association. These ideas align with the suggestion that a key function of the peer-to-peer reciprocity of juvenile social play fighting is to support social skills, emotional regulation, adaptability and resilience [32,33] through impacting the mPFC-mediated development of executive function skills, such as flexibility and inhibition [32,91]. However, while much of the evidence linking play levels to flexibility involves social play or a lack thereof, further studies on locomotor and object play interventions are required. For example, playing with objects may help individuals learn the ways in which they can interact with future, similar objects (i.e. moving from ‘What can this object do?’ to the question of ‘What can I do with this object?’ [96]), while locomotor play may allow individuals to learn the ways in which they can use their body.

In humans, one study reported that positive parental attitudes on the importance of play for child development were associated with better task-based flexible shifting in their children, but only in a Hungarian and not an Ethiopian sample [97]. As such, opportunities to play facilitated by parents may support task-shifting flexibility, although the reasons for the cross-cultural differences are unclear. Additionally, greater retrospectively reported freedom in play opportunities in childhood was shown to be associated with adulthood social success, mediated by flexibility in adjusting personal goals [98]. The authors suggested that the diversity of childhood play facilitates the ability to adapt values, attitudes and goals to environmental changes throughout life, enhancing success [98], although success may also enhance the positivity of an individual’s view of their childhood.

Other studies have examined the impact of play opportunity interventions on flexibility outcomes in children, addressing the limitations related to cross-sectional studies with self-reported play assessments. An eight-week pretend play intervention [99], a single 9 min physical play intervention session [100] and a seven-week intervention with both adult- and child-directed role play [101] all supported children’s cognitive flexibility (i.e. shifting between the use of different rules when sorting cards). However, these studies assessed cognitive flexibility at the end of the intervention and did not examine the maintenance of these effects. Furthermore, two other studies did not identify an effect of play interventions on cognitive flexibility when using a six-week ‘dramatic play’ intervention involving acting out different roles [102] and a five-week intervention involving ‘fantastical’ play (i.e. acting out different roles facilitated by adults) or non-imaginative play with balls or games [103]. This heterogeneity may be explained by factors such as the depth of the intervention, the time elapsed between treatment and tests, the cognitive flexibility measures, home play environments and the types of play encouraged.

### (b) Play as a contributor to resilience

#### (i) Playfulness trait and resilience

In the human literature, certain studies have highlighted associations between playfulness and resilience in response to stress [6]. Specifically, it has been suggested that the experiences an individual has during childhood play may help them to learn to overcome uncertain or difficult situations [104,105]. Furthermore, in line with the broaden-and-build theory, the increased thought–action repertoires resulting from the positive affect generated by being playful may give the individual the flexibility to identify and implement a range of coping strategies to use across situations [6].

Regarding experimental evidence, various studies have reported relationships between playfulness and resilience using self-reports. For instance, self-reported playfulness, measured based on characteristics such as being ‘energetic’ and ‘spontaneous’, was found to relate to self-reported lower stress and the use of effective coping styles [106]. Similarly, self-reported playfulness on the short measure of adult playfulness (SMAP) [107] was reported to relate to self-efficacy and coping during the COVID-19 pandemic [108]. Finally, increases in self-reported playfulness on the older adult playfulness scale [109] were found to relate to the growth in women’s self-reported resilience [110].

These human studies suggest a connection between playfulness and resilience, although similar studies in animals are lacking. The impact of self-report bias should be considered, whereby those who see themselves as more playful are also more positive about their coping. However, it could also be argued that perhaps an individual’s self-perception of their coping abilities is what matters. Furthermore, as mentioned at the beginning of §2, playfulness in humans has been measured using varying scales, increasing methodological heterogeneity and making this evidence more difficult to apply to ‘playfulness’ as a personality trait in animals.

#### (ii) Play levels and resilience

Multiple studies have assessed the impact of play levels on resilience in animals through the paradigm of social play deprivation. In rats, one study reported that social play deprivation impacted their ability to show appropriate responses to an aggressor later in life, leading to them sustaining more injuries and emitting more distress vocalizations [111]. In non-aggressive contexts, these isolated rats engaged in fewer stress coping behaviours, including play and social grooming [111]. Social play deprivation in hamsters was reported to cause inappropriate aggression, increased conditioned defeat (i.e. fear conditioning involving consistent submissive responses to an aggressive conspecific), and avoidance of familiar opponents, interpreted as them showing increased vulnerability to stressful social situations in adulthood [112]. Interestingly, 1 h of play encounters per day was found to rescue these effects on conditioned defeat and prevent increased agonistic behaviour in males caused by social play deprivation [113]. Individuals deprived of play may simply prefer to avoid agonistic interactions or learn about their consequences faster, but their engagement in inappropriate aggression when not defending their home territories makes this less likely.

It should be remembered that the play deprivation method limits all social contact during the juvenile period, meaning the results may be confounded by social isolation [114]. However, some studies have addressed this by providing the isolated group with a short daily period of play [91] (see §2a(ii)). Additionally, outside of the play deprivation paradigm, a study in captive marmosets reported that those who received more social play behaviour solicitations from family members following a period of separation showed better HPA recovery (based on the return of urinary cortisol to baseline levels) the day after [115], suggesting better resilience to this stressor.

## 3. Recommendations for approaching the play–welfare relationship in captive animals

The theories and findings reviewed here support the case for play building flexibility and resilience to stress in captive animals, as highlighted in the model in figure 1. If play contributes to the development of long-term flexibility and resilience, captive animals subject to limited play opportunities by intensive management systems may be especially susceptible to negative welfare and poorer resilience to common stressors. Consequently, methods for enabling the expression of play and how they impact affective state, flexibility and resilience must be investigated. Indeed, it has already been suggested that play opportunities, which often involve the provision of play objects, conspecifics to play with or extra space in captive animals, could be explored as a way to enhance the environment and improve welfare by enhancing autonomous control [116,117], improving behavioural competence (e.g. [71]), reducing boredom [118,119] and providing rewarding learning experiences [16].

### (a) Using play to enhance welfare

In children, play therapy is efficacious for treating stress, behavioural issues and psychological distress [120], indicating that play-based interventions improve affective state and coping with stress. In adults, humour is considered an element of playfulness [84], and humour-based therapy has been shown to reduce depression and anxiety (for a review, see [121]). Indeed, in patients with schizophrenia, an intervention involving humour skills training and practising free ‘home-play’ reduced symptoms of anxiety and depression [122].

Translating to animals, the predictions of the model in figure 1 linking opportunities to play to the development of flexibility and resilience suggest that applying changes to the way in which captive animals are housed to enable play should represent a positive change for welfare. For example, adequate space and comfortable, stable flooring need to be available for individuals to run around, pain management needs to be used effectively to ensure play is not suppressed, and environments could be made more complex through the provision of appropriate objects and spaces. Pigs have been shown to engage in more locomotor and object play when provided with straw bedding daily [123], and providing preferred objects to calves, such as brushes, teats, pipes with molasses and hanging ropes, increased locomotor play [124]. A study in elephants [125] reported that play increased when they were provided with outdoor space overnight, and giving enrichment objects to farmed peccaries (e.g. a ball, hose and see-saw) increased locomotor and social play [126]. Regarding outcomes of increased play opportunities, rats have been shown to produce more 50 kHz vocalizations when spending time in a ball pit or playpen compared to the home cage, indicating a positive affective state [127], and playpen access reduced the negative affective impact of an anxiogenic drug in an affective bias test [127]. In ferrets, positive outcomes of ‘playtime’ (aiming to stimulate object, locomotor and social play) have been documented for reducing boredom-like behaviour [128]. In gorillas, sessions involving play with handlers resulted in positive behavioural changes, including reduced stereotypic behaviour, aggression and inactivity and increased affiliative and playful behaviour [129]. Finally, providing laboratory mice with a ‘playpen’ (i.e. larger cages with increased complexity) for 30 min three times per week reduced aggression and anxiety-like behaviour [130]. However, these individuals also showed increased stereotypies in the home cage, which are often thought to indicate a more negative affective state, potentially due to a negative contrast with the playpen [130].

These studies highlight the positive impacts of additional opportunities to engage in all types of play on affective state. However, given the predictions of the model in figure 1, it would be interesting to additionally explore the specific impacts of play provision interventions on flexibility and resilience and how long such impacts last. It should also be considered whether certain factors moderate the effects of increased play levels on welfare, such as the species, the type of play encouraged, the age of the individual and the location and duration of interventions.

#### (i) Considerations when using play to enhance welfare

First, play provision may differentially impact animals of different species. A significant amount of research on play in captive species has focused on laboratory rats, for which social play during the juvenile period is well characterized (e.g. [32]). Other captive species for which play during the juvenile period has been well documented include cattle (e.g. [81]), pigs (e.g. [131]), sheep (e.g. [132]) and chickens (e.g. [133]), where play behaviours have been used as a welfare indicator. However, this focus does not preclude play being important for other, less common captive species. For example, there are suggestions of object play in birds (e.g. [134]), fish (e.g. [135]) and octopus (e.g. [136,137]), but perhaps not enough is known about their play to understand their motivation for different play types and what environmental characteristics may enable play.

Second, further work is warranted to understand the potentially varying impact of encouraging different play types. Providing object play opportunities may not have the same impact as providing, for example, space for locomotor play or appropriate peers for social play, especially given their partially dissociable motivational underpinnings [72,73]. While playing with an object may support an individual to gather information on how they can flexibly interact with objects in their environment, social play may be more useful for developing the flexibility to manage various social situations [3]. Restrictive captive systems that stop individuals from running around, do not allow them space to play fight or do not provide enrichment objects potentially provide a model for investigating how increasing the opportunity for each type of play affects long-term outcomes, although cleanly manipulating just one type may not be straightforward.

Third, play interventions that are carried out for limited durations or outside of the home environment may have different effects on welfare compared to permanent changes to the home environment that facilitate meaningful opportunities to play throughout an animal’s life. For example, play interventions given outside of the home environment may generate a negative contrast when the individual is returned to a barren, restrictive pen or cage. For this reason, laboratory mice in [130] may have shown greater stereotypies in the home cage when they were used to having access to an external playpen for 30 min per day. If interventions are short-lived and only given during the juvenile period, this may also miss the proposed immediate benefits of providing play opportunities to adults in barren environments. Human adults spontaneously play when they are bored at work [138], and social play in adult animals could be important for ensuring appropriate social dynamics (e.g. [26,34]) and reducing social stress or boredom (i.e. a negatively valenced state caused by understimulation [139,140]). Additionally, giving increased play opportunities during only the juvenile period could create a detrimental contrast between the animal’s rearing environment and its adulthood reality. Indeed, an animal with enhanced play opportunities with its peers during the juvenile period may be better able to deal with dynamic social interactions in adulthood but may not have the tools to manage boredom in a barren and unchanging environment. In fact, developing skills (e.g. mastering a complex environment) that will not be useful in a future barren environment may even be disadvantageous due to negative contrast and frustration resulting from unmet expectations.

Finally, the relationship between play levels and welfare itself is not linear. Increased playfulness can accompany stress and negative affect [26], although it could be argued that play itself is being used as a mechanism of balancing negative and positive affect and managing such difficulties. It was also found that mice that were more playful as juveniles showed higher levels of anxiety-like behaviour and lower exploration later in life [77], potentially indicating that being playful may not always be overwhelmingly positive. Indeed, in humans, it has also been suggested that there are ‘darker sides’ of playfulness, such as having lower restraint and being more risk-seeking [6].

### (b) Exploring playfulness as a trait in animals

In animals, personality traits such as playfulness are defined by the stability in the behaviour across time and contexts (i.e. an individual showing a consistent propensity to play) [85,141]. In humans, Shen *et al*.’s [83] conceptualization of playfulness includes the dimensions of fun-seeking motivation, uninhibitedness and spontaneity, and Proyer [82] presented four playfulness dimensions: ‘other-directed’ (e.g. enjoying playing with others), ‘light-hearted’ (e.g. being easy-going), ‘intellectual’ (e.g. playing with ideas, preference for complexity) and ‘whimsical’ (e.g. preference for things that are unusual). Additionally, a children’s measure of playfulness [142] focuses on humour and joy, flexibility and imagination, physical activity and social competence during play. In the future, studies could explore the nature of the playfulness trait across species by examining the stability in play levels and characteristics across time and contexts [141] and associations with these dimensions from the human literature, including play vigour [142], how the individual plays with others (e.g. initiating and maintaining play) [142] and their motivation to access play [83]. Some human playfulness dimensions seem more tractable than others for measurement in animals. For example, flexibility and social play competence may be easier to operationalize than imagination or humour.

If the playfulness trait were confirmed more broadly, studies could explore the association between playfulness and flexibility (e.g. task-based reversal learning, generating novel actions) or resilience to stressors (e.g. speed of recovery following a challenge). However, finding an association between playfulness and these outcomes would not necessarily imply causation; for this, it may be more useful to focus on the impact of supporting playfulness by providing extra opportunities to play. Indeed, in humans, a playfulness intervention had positive effects on playfulness, wellbeing and depression [87]. Another issue with investigating the relationship between playfulness and task-based cognitive flexibility, such as reversal learning (commonly used as a flexibility measure in experimental psychology), is that the playfulness trait in humans is associated with uninhibitedness [83], meaning playfulness may make the inhibition of previous responses in these tasks more difficult. Furthermore, reversal learning performance may be a distinct skill compared to innovation (e.g. obstacle removal in foraging tasks), which has been used as a measure of behavioural flexibility in behavioural ecology [37]. Therefore, when linking flexibility and playfulness in animals, flexibility assessments must be chosen with careful consideration of the skills they are measuring.

## 4. Conclusion

This work explored the role of play behaviour in contributing to captive animal welfare through the development of flexibility and resilience. The training for the unexpected theory [39] and the broaden-and-build theory of positive emotions [53,54] suggest that play may support the development of flexibility through providing both opportunities to learn about different ways to respond to changes in the environment and broadening thought–action repertoires, respectively. This enhanced flexibility should then support the resilience of captive individuals when they are faced with inevitable challenges. Although limited in number, some studies provide findings suggestive of a relationship between play behaviour and flexibility- and resilience-related outcomes in humans and animals and the potential neural bases of such associations. Consequently, this review suggests that play, especially in captive animals, should be understood and further explored as an important contributor to welfare through its potential role in supporting the development of flexible, resilient individuals. In this context, appropriate and effective methods for enabling the expression of play in captive animals must be investigated, alongside the impact of play opportunity provision on outcomes such as affective state, flexibility and resilience. Consideration should also be given to whether time-limited interventions, as opposed to permanent, meaningful changes in the housing environment, are always positive for welfare depending on their timing and duration. Such evidence will be useful for formulating more specific recommendations regarding the types of play opportunities required by captive individuals and how to provide them. Finally, since playfulness is conceptualized as a trait in humans that is associated with positive outcomes, future studies should investigate the playfulness trait and its correlates in non-human animals.

## Data Availability

This article has no additional data.
